# *ApoB* gene polymorphism (rs676210) and its pharmacogenetics impact on atorvastatin response among Iraqi population with coronary artery disease

**DOI:** 10.1186/s43141-021-00193-4

**Published:** 2021-06-22

**Authors:** Shaimaa Y. Abdulfattah, Salwa J. Al-Awadi

**Affiliations:** 1 Biotechnology Research Center, Al-Nahrain University, Baghdad, Iraq; 2 College of Biotechnology, Al-Nahrain University, Baghdad, Iraq

**Keywords:** Atherosclerosis, *ApoB* gene, Coronary artery disease, Genetic, Single nucleotide polymorphism

## Abstract

**Background:**

Drug response is below genetic influence, proven by the genetic variants. Pharmacogenetics trials are performed in many diseases, including coronary artery disease. This study was designed to determine the genetic polymorphism (rs676210) Pro2739leu G > A in the lipid metabolism-related gene (*ApoB* gene) and its pharmacogenetic role in the response to atorvastatin drug in a sample of Iraqi population with coronary artery disease (CAD).

**Results:**

Significant differences of genotype distribution in CAD patients and controls were observed in *ApoB*_+ 8216_ in Iraqi population from Hardy Weinberg Analysis. It also found that dramatic difference of low-density lipoprotein (LDL-C) level in response to 40 mg/day of atorvastatin therapy, the minor allele (*A*) observed a greater LDL-C lowering than the wild type allele (G). In ANOVA analysis, the result showed that the rs676210, Pro2739Leu, in *ApoB* gene increased non significantly, but gradually in plasma level of total cholesterol (TC), triglyceride (TG), very low-density lipoprotein (VLDL), and oxidize low-density lipoprotein (oxLDL) in the order of genotype AA, GA, and GG in response to 40 mg atorvastatin.

**Conclusion:**

We found the results highlighted the function of the rs676210, Pro2739Leu, in the *ApoB* gene in CAD etiology, and the findings support this variant’s impact in predicting the response of (LDL-C) to 40 mg of atorvastatin therapy. *ApoB* gene polymorphism (rs676210, Pro2739Leu), specifically the AA genotype, may help to identify individuals who will profit from atorvastatin's lowering effects.

## Background

Coronary artery disease (CAD) is an arterial disease complex, involving medium and large arteries comprising various genetic and environmental conditions [1]. It is categorized by vascular inflammation, endothelial dysfunction, and lipid accumulation such as cholesterol in the intima region of the vessel wall [2]. This construction results in a plaque deposition, which accumulates on the internal walls of the arteries and is generally referred to as a hardening or furring of the arteries, and as the artery walls thicken, the blood stream narrows, and this can reduce or obstruct the blood flow which reduces the supply of oxygen to target organs. Various pathogenesis changes in biological properties including elevated macrophage absorption and chemical activation of monocytes in the arterial walls and increased chance of plaque rupture was associated with excessive levels of ox-LDL [3]. According to the World Health Organization (WHO), cardiovascular disease (CVD) is an important cause of morbidity and mortality in developed countries and the risk of death from cardiovascular disease is rising and 23 million people will die each year in 2030 [4]. Etiological, genetic, and environmental factors have just been documented to contribute to the enhancement of CAD and among genetic factors, genes linked to lipid metabolism such as (*LDL-R*, *ApoB*, and *PCSK9* genes) have been documented to have a major effect on the disease predisposition. In the past several years, some atherosclerosis researchers have been trying to identify predisposing genotypes that concentrate primarily on single nucleotide polymorphisms (SNPs) that play a critical role in lipid metabolism [5].

Pharmacogenetics is the pharmacology field that struggles with the genetic effect variation on drug response in patients through the related gene expression or SNP with the activity or sensitivity of a medication. It is objected that reasonable means built to customize drug with regard to the patient's genotype, to ensure full effectiveness with limited adverse effects [6]. The basic elements of lipoprotein particles are apolipoprotein, which act as ligands of lipoprotein receptors and lipid metabolizing enzymes. A major structural protein of chylomicron (VLDL and LDLc) is apolipoprotein B (ApoB) [7]. Several genetic variations or polymorphisms were found within this gene, and such a polymorphism was identified in the *ApoB* gene associated with LDL particle oxidation was rs676210 SNP c. 8216G > A (Pro2739Leu). This SNP presents in exon 26 causing a proline to leucine substitution which was expected to trigger a functional change in the protein structure of ApoB [8].

Genetic variations in the *ApoB* gene may lead to an increased the level of LDL particles and other lipid metabolic disorders, as well as an increased risk of coronary artery disease. The elevated serum ApoB levels, even with a normal level of LDL, often cause hypobetalipoproteinemia, normotriglyceridemic hypobetalipoproteinemia, and hypercholesterolemia [9, 10]. ApoB-100 is not only a structural component, but also a ligand for LDL receptor that mediated the endocytosis. Essentially all circulating ApoB are connected to lipoproteins, and unlike most the other apolipoproteins, ApoB cannot freely exchange with other lipoprotein particles [8, 11]. Atorvastatin (3R, 5R)-7-{2-(4-fluorophyenyl)-3-phenyl-4 (phenylcarbamoyl)-5-propan- 2-ylpyrrol-1-yl}-3,5-dihydroxyhepatonic acid (LIPITOR) is abroad used a statin therapy to lower the serum lipid level with a better safety and tolerance and was patented approved in 1986 for medical use in the USA in 1996 [12]. Atorvastatin is a class of statins used in Iraqi healthcare services and two doses 20 mg/day and 40 mg/day were dependent in the present study [13].

This study was implemented in an Iraqi patient to explore the function of the genetic variability of *ApoB* gene with respect to atorvastatin’s lipid-lowering effect. In this research, we investigate the role of rs676210, Pro2739leu, G > A polymorphism in the *ApoB* gene in responding of lipid profile and oxLDL levels to atorvastatin drug with two doses (20 and 40 mg/day). The choice of the selected *ApoB* gene SNP (rs676210) has been focused on their physiological role in lipid metabolism, LDL-C oxidation, and cardiovascular health based on the most previous publications [8, 14].

## Methods

### Study design

The existing case-control study has been authorized by the ethics committee of the Iraqi Ministry of Health, including 100 CAD patients attended to the Iraqi center of heart disease in Ghazi Al-Hariri Hospital/Baghdad, Iraq, for diagnosis and treatment during the period from June 2018 to March 2019. The diagnosis is based on WHO guidelines including chest pain with either electrocardiographic (ECG), echocardiography, treadmill test (TMT), or a full clinical history of myocardial infarction (MI); angina pectoris (stable and unstable) has been requested [10]. All patients received atorvastatin drugs, and the patients were distributed into two clinical subgroups according to drug dose. The first includes 52 patients treated with 20 mg/day and the second includes 48 patients treated with 40 mg/day. Patients will be registered if they achieve any of the following inclusion criteria were patients diagnosed as (CAD) with TC (usually > 200 mg/dL, LDL-c > 160 mg/dl, and TG < 200 mg/dl) according to the American Heart Association (AHA) guidelines and normolipidemic subjects with LDL-c < 130 mg/dl and TG < 150 mg/dL, and their age ranged from 35 to 60 years.

#### Exclusion criteria

Subjects with hypertension (HT) were described as (1) previously, the patient had diagnosed with HT by a clinician, or (2) systolic blood pressure equivalent to 140 mmHg and/or diastolic blood pressure equivalent to 90 mmHg on two separate occasions. Diabetes mellitus has been identified as fasting blood glucose treatment of up to 126 mg/dl. Hypertriglyceremia; liver, renal, or thyroid disease; malignant tumor; autoimmune disease; congenital heart disease (CHD); and patients with inadequate basic details were omitted to prevent the findings from being inaccurate. The control group included 100 Iraqi subjects with healthy levels of cholesterol and not documented with CAD. Upon entering the study, the aims of the study and the medical ethics signed were clarified to both patients and control. Each subject’s demographic characteristics were documented by analyzing the answered questionnaire via a direct interview with patients and control groups.

### Biochemical assays

After 12 h of fasting, blood samples were taken in the morning. Serum samples were tested using enzymatic methods for TC, LDL-C, HDL-C, and TG tests and was carried out using Biolabo reagents (Biolabo/France). Serum low-density lipoprotein (LDL-C) was calculated by Friedewald’s formula. The concentrations of oxLDL were determined from thawed serum using the Mercodia Oxidized LDL ELISA 10-1143-01/LOT 29196 (Mercodia, Uppsala, Sweden) which is a solid-phase immunoassay adapted from the original Holvoet method. It is directed against a conformational epitope in the apolipoprotein B100 moiety of LDL produced as a result of the conversion of at least 60 apolipoprotein B100 lysine residues with aldehydes. The concentration of oxLDL in each serum sample was expressed in U/L for comparison of results with those of controls concentration and detection ranges 26–117 U/L [15].

### Genetic analyses

**Fig. 1:**
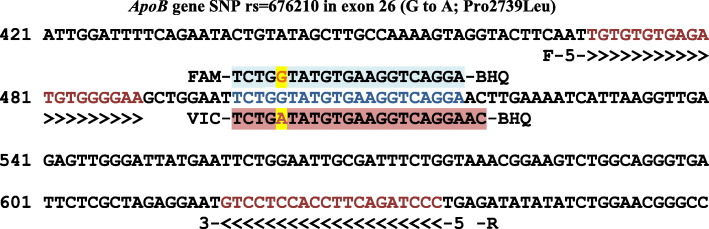
Matching of the primer and probe sequences of *Apo B* gene in Exon 26 on the bioinformatic programs/NCBI

The genomic DNA from EDTA blood was purified by the use the Wizard Genomic DNA Purification Kit (Promega, USA). This research investigated ApoB gene polymorphism (rs676210) G > A present in the coding region, exon 26, and causes a missense mutation at position 2739 substitutes the amino acid proline to amino acid lysine. The polymorphism was examined by the thermal profile of allelic discrimination methods used real-time PCR (RT-PCR) (Cephied, USA). TaqMan fluorescent oligonucleotide probes and primer sequences were designed according to their reference sequences (rs) in the database of NCBI (National Center for Biotechnology Information) and synthesized by Alpha DNA Ltd (Canada) and stored at − 23. The real-time PCR was performed in a total volume of 25 μl containing 0.5 μl of forward and reverse primer, 0.5 μl for each FAM and VIC probe, TaqMan master mix (Promega, USA) 12.5 μl, PCR grade water 6.5 μl, and Genomic DNA 4 μl. The thermal profile of allelic discrimination real-time PCR is initial denaturation at 95 °C for 6 min was followed by 5 cycles of denaturation at 95 °C for 25 s, annealing at 60 °C for 25 s, and extension at 72 °C for 20 s, and then followed by 35 cycles of denaturation at 95 °C for 20 s, annealing at 59 °C for 30 s, and extension at 72 °C for 20 s. A reporter dye (FAM, VIC) at the 5′ end and a quencher dye (BHQ) at the 3′ end were included in the TaqMan probe. The sequence of each probe and primer used in the allelic discrimination is shown in (Table 1; Fig. 1).

**Table 1:** Primers and probes of *ApoB* gene polymorphism (rs676210) used in the study

Primer/probes	Sequence (5′-3′)	Product size, bp
**Forward**	5′-TGTGTGTGAGATGTGGGGAA-3′	167
**Revers**	5′-GGGATCTGAAGGTGGAGGAC-3′	
**FAM-Probe**	5′-TCTGGTATGTGAAGGTCAGGA-3′-BHQ	
**VIC-Probe**	5′-TTCTGATATGTGAAGGAAC-3′-BHQ

### Statistical analysis

The alleles and genotypes of ApoB SNP have been shown as a percentage, frequency distribution, and significant differences between their distributions in patients with CAD, and controls were tested by Fisher’s exact probability (p). In addition, odds ratio (OR) and its 95% (CI) were also calculated to define the association between allele or genotype and CAD. Analysis was achieved by using the WINPEPI program version 11.36. All ApoB SNP frequencies were estimated using a system of direct gene counting, while a major deviation from Hardy-Weinberg equilibrium (HWE) was estimated using the HWE calculator for two alleles. Pearson’s chi-square test has measured significant differences between the observed and expected genotype frequencies. Continuous variables in the data were expressed as mean ± standard deviation (SD).

## Results

### Demographic distribution of the study population

In the previous of our publication, some demographic characteristics were studied and demonstrated. However, the mean age, gender, weight, height, BMI, SBP, DBP, and drinking habit differed non significantly between the two study groups (*P*< 0.05; Table 2) [16].

**Table 2:** Demographic characteristics of the study groups

Variable	Control (***n*** = 100)	Patients (***n*** = 100)	***P*** value
Age (year)^a^	46.67 ± 9.25	48.81±8.21	0.10 (NS)
Gender^b^			0.40 (NS)
Female	26 (26 %)	21 (21 %)	
Male	74 (74 %)	79 (79 %)	
Weight (kg)^a^	80.34 ± 13.42	81.41 ± 12.47	0.56 (NS)
Height (cm)^a^	172.96 ± 9.00	170.37 ± 8.38	0.59 (NS)
BMI (kg/m^2^)^a^	28.06 ± 3.38	28.18 ± 4.20	0.84 (NS)
Systolic pressure (mmHg)^a^	110.89± 14.03	120.21±13.98	0.29 (NS)
Diastolic pressure (mmHg)^a^	70.45 ±12.00	80.32 ± 13.79	0.21 (NS)
Smoking Status^b^			**0.02**
Non smoker	76 (76 %)	61 (61 %)	
Smoker	24 (24 %)	39 (39 %)	
Total	100 (100%)	100 (100%)	
Alcohol status^b^			0.12 (NS)
Consumed	0 (0 %)	4 (4 %)	
Non consumed	100 (100 %)	96 (96 %)	
Total	100 (100%)	100 (100%)	

Data were expressed as counts with percentages in parentheses or mean ± SD

*NS* no significant differences; significant values are bolded

^a^Statistical analysis was performed by independent t test

^b^Statistical analysis performed by the chi-squared test

### Distributions of genotype and allele frequency

The *ApoB* gene SNP at position + 8216 (ApoB_+ 8216_) was identified in two studied groups with three genotypes (GG, GA, and AA) corresponding to two alleles (*G* and *A*). HWE analysis showed a significant difference in the control group between the observed and expected genotype frequencies and was deviated from HWE; no significant difference was found between the observed and expected genotype frequencies in the patient group and was consistent with HWE (Table 3).

**Table 3:** Number and percentage frequencies (observed and expected) of *ApoB* gene (rs676210 SNP) genotype and their Hardy-Weinberg equilibrium (HWE) in patients and controls

Genotype	Control (No. = 100)		Patient (No. = 100)	
	Observed	Expected	Observed	Expected
	No (%)	No (%)	No (%)	No (%)
**GG**	42 (42)	34.81 (34.81)	66 (66)	64 (64)
**GA**	34 (34)	48.38 (48.38)	28 (28)	32 (32)
**AA**	24 (24)	16.81 (16.81)	6 (6)	4 (4)
**HWE analysis**	*χ*^2^ = 8.83, DF = 1, *P* ≤ 0.01		*χ*^2^ = 1.05, DF = 1, *P* > 0.05	

1 degree of freedom (d.f) for chi-squared distribution

The genotype (GG) showed a statistically significant difference in the control group compared to the patient group (42% vs 66%; OR = 2.8; 95% CI 1.52–4.74; *P* = 0.001; EF = 0.41). Also, there was a significant difference observed in the mutant genotype (AA) between the two investigated groups (24% vs 6%; OR = 02; 95% CI 0.08–0.52; *P* = 0.0005; PF = 0.19). It was also observed that the mutant allele (A) revealed a significant increase in its frequency in the control group when compared with the patient group (41% vs 20%; OR = 0.36; 95% CI 0.23–0.56; *P* = 0.0007) with a protective effect (PF = 0.26). In addition, there a decrease frequency was observed in the (*G*) allele in the control group compared to patient group (59% vs 80%; OR = 0.2.78; 95% CI 1.78–4.34; *P* = 0.0007) with an etiological effect (EF = 0.51). No significant differences were revealed in heterozygous genotype (GA) of the two investigated groups (34% vs 28%; OR = 0.75; 95% CI 0.41–1.37; *P* = 0.44; PF = 0.083) (Table 4).

**Table 4:** Genotype and allele frequencies of *ApoB* gene polymorphism rs676210 between patient and control groups

Groups	***ApoB*** gene at position + 8216 (dbSNP-ID: rs676210)				
	Genotypes			Alleles	
	GG	GA	AA	*G*	*A*
Controls (No. = 100)					
No.	42	34	24	118	82
%	42%	34%	24%	59%	41%
Patients (No. = 100)					
No.	66	28	6	160	40
%	66%	28%	6%	80%	20%
(OR)	2.68	0.75	0.2	2.78	0.36
(EF) or (PF)	0.41	0.083	0.19	0.51	0.26
P	0.001	0.44	0.0005	0.0007	0.0007
95% (CI)	1.52-4.74	0.41-1.37	0.08 – 0.52	1.78-4.34	0.23-0.56

*OR* odds ratio, *EF* etiological fraction, *PF* preventive fraction, *P* Fisher’s exact probability

### Pharmacogenetic effects of rs676210 SNP on lipid-lowering response to atorvastatin

The serum lipid levels and ox LDL in control and patients with 20 mg and 40 mg subgroup according to rs676210 genotype are shown in Tables 5, 6, and 7, respectively. There were no significant variations in the parameters of lipid profile and the level of oxLDL between the three genotypes of the control group.

**Table 5:** The impact of the *ApoB* rs676210 polymorphism on the lipid profile and oxLDL of the control group

Parameters	Genotype (mean+SD)			
	G/G (***n*** = 66)	G/A (***n*** = 28)	A/A (***n*** = 6)	***P*** value^**‡**^
TC (mg/dl)	140.52±20.01	136.83±26.26	134.02±15.43	0.63
TG (mg/dl)	111.63±17.11	113.47±20.62	115.08±12.49	0.84
HDL (mg/dl)	52.21±9.47	51.49±8.77	49.25±13.35	0.74
LDL (mg/dl)	65.98±19.79	62.65±23.39	61.75±24.49	0.73
VLDL (mg/dl)	22.32±3.42	22.69±4.12	23.01±2.49	0.84
OX-LDL (U/L)	50.20±8.77	43.39±9.66	38.58±12.90	0.42

^‡^ANOVA significance test (2-tailed)

**Table 6:** Lipid profile and oxLDL response to atorvastatin treatment (20 mg/day) according to apolipoprotein B (*ApoB*) rs676210 polymorphism in the patients with CAD

Parameters	Genotype (mean+SD)			
	G/G (***n*** = 24)	G/A (***n*** = 13)	A/A (***n*** = 15)	***P*** value^**‡**^
TC (mg/dl)	270.71±78.63	294.11±52.64	273.81±78.97	0.76
TG (mg/dl)	151.14±41.61	145.00±31.37	156.36±45.49	0.83
HDL (mg/dl)	50.71±3.40	48.22±1.78	46.63±2.76^b^	**0.01**
LDL (mg/dl)	195.81±72.95	216.88±47.06	208.78±66.65	0.80
VLDL (mg/dl)	30.22±8.32	29.00±6.27	31.27±9.09	0.82
oxLDL (U/L)	82.90±29.01	78.72±18.24	77.58±26.16	0.90

^‡^ANOVA significance test (2-tailed)

^b^*P* ˂ 0.05 GG group vs. AA group

**Table 7:** Lipid profile and oxLDL response to atorvastatin treatment (40 mg/day) according to apolipoprotein B (*ApoB*) rs676210 polymorphism in the patients with CAD

Parameters	Genotype (mean+SD)			
	G/G (*n* = 18)	G/A (*n* = 21)	A/A (*n* = 9)	*P* value^‡^
TC (mg/dl)	404.33±183.85	339.96±73.24	298.11±54.68	0.14
TG (mg/dl)	194.22±97.28	177.37±51.28	154.55±25.19	0.41
HDL (mg/dl)	48.22±5.09	48.60±5.70	51.11±5.48	0.46
LDL (mg/dl)	325.62±84.21	261.88±63.04	219.37±54.27^b^	**0.008**
VLDL (mg/dl)	38.84±19.45	35.47±10.25	30.91±5.03	0.41
oxLDL (U/L)	71.25± 17.80	63.24± 20.95	58.13±18.29	0.25

^‡^ANOVA significance test (2-tailed)

^b^*P* ˂ 0.05 GG group vs. AA group

The findings in patients with 20 mg atorvastatin examining the response to atorvastatin treatment and showed no significant difference in TC and LDL levels, but there was a significant difference in HDL and reported the highest level of GG genotype (50.71±3.40) compared with AA genotype (46.63±2.76) *P* = 0.01.

Results in patients with 40 mg atorvastatin showed a strong correlation between AA genotype and LDL-C level in response to 40 mg/day atorvastatin therapy, thus the homozygous minor allele (*A*) reported a greater reduction in LDL-C than individuals carrying wild-type homozygous (*G*) alleles. The result showed the (rs676210, Pro2739Leu) in *ApoB* gene increased gradually, but non significantly in plasma level of TC, TG, VLDL, and oxLDL in the genotype order of AA, GA, and GG in response to 40 mg atorvastatin.

## Discussion

In this study, we shed light on the impact of ApoB (rs676210) gene polymorphism on the response of atorvastatin drugs and the frequency of alleles in the Iraqi population. In the current study, it has been noticed that SNP rs676210 has a putative effect on Apo B protein structure of LDL particle, and the SNP may be acting as a protective factor for atherosclerosis coronary condition in individuals who carrying AA and GA genotypes in comparison of GG genotype that has a high risk of the disease. So, the *A* allele has a protective effect against disease development (*A* allele: OR = 0.36) while the *G* allele can be regarded as a predisposing allele in the ApoB_+8216_ (rs676210) SNP (*G* allele: OR = 2.78). The findings reported were consistent with positive and negative associations between ApoB_+8216_ SNP (rs676210) and CAD etiopathogenesis, and some researchers were also in support of this study. In two earlier studies on Chinese Yugur populations showed a positive correlation between *G* allele and GG genotype with CAD (susceptible allele) while *A* allele frequency was (protective allele) and *G* allele of rs676210 had a significantly higher risk of MI and may result in elevated rates of ApoB and LDL-C plasma in the control subjects [8, 10].

Our observation revealed a deviation from the HWE analysis in the control group between the observed and expected genotype frequencies. In this regard, it has been well reported that the allele frequencies of ApoB SNP (rs676210) display deviations across Iraqi populations and the study had a relatively limited sample size as a limitation factor. For rs676210 distribution of genotype and allele frequencies, there was a notable difference between control and patients (*A* allele frequency: 41% vs. 20%; OR = 0.36; *P* = 0.0007) in the present study. By evaluating the literature, [17] published a study which found that the three genotypes of rs676210 were observed with a significant frequency in the population of Caucasians. Such observation suggests by [14] demonstrated the significant difference between *G* allele and *A* allele of rs676210 in a cohort study of patients in the Finland population. In a cohort study in Sweden, the frequency of rs676210 SNP in the alleles and genotypes between patient and control was significantly increased [18]. Certain pathogenic and etiological functions of oxLDL can be understood when determining the *ApoB* rs676210 polymorphism. In the current study, we found that the mutant AA genotype of rs676210 was related to the regulation of oxLDL level. The genome-wide association analysis (GWAS) showed that rs676210 is an important factor in controlling the levels of oxLDL. The explanation why rs676210 is associated with oxLDL regulation may be that the ApoB (GG) genotype allows LDL particles high susceptible to oxidation in the intima arteries and the SNP in homozygous minor allele (*A*) carriers. The minor allele *A* of rs676210 resulted in amino acid substitution of proline to lucine, which could further change the ApoB moiety and the 3D configuration making the LDL particles less sensitive to oxidation [19]. A previous suggestion revealed that the LDL is easier to store in the intima matrix, so that the LDL molecule may be exposed to oxidative processes longer [14]. The findings obtained support such interpretation on the base of its pharmacogenetic effects, AA genotype showed a greater lowering of LDL-C than did individuals bearing the wild type allele (*G*) in patients with 40 mg of atorvastatin (219.37±54.27 vs. 325.62±84.21: *p* = 0.008). A previous research by [20] showing that the minor allele (*A*) of rs676210 was linked with lower levels of TG, TC, and LDL and higher levels of HDL cholesterol in response to statin therapy compared to major (*G*) carriers with a *P* < 5×10−8. Amusingly, [17] found that the mutant allele (*A*) of rs676210 was correlated with enhanced fenofibrate treatment response and decreased LDL oxidation, and the therapy was identified to lower TG levels by “24.7%, 28.3%, and 34.5% according to the genotypes GG, GA, and AA of rs676210 SNP respectively.” This SNP was also studied and approved for the TC-reduction efficacy and decreased after 40 mg simvastatin treatment [21].

A prior examination carried out a similar finding in a further study of [22] who observed the *A* allele was correlated with lower levels of TC, LDL, TG, and higher level of HDL as opposed to the (*G*) allele. On the other hand, patients bearing minor alleles of the other genes such as ABCA1 (rs12003906) and APOE (rs7412) SNPs increased their reduction in LDL-C in the highest doses of atorvastatin [23]. A contradictory finding was observed in patients with growth hormone deficiency (GHD) in Sweden, ApoB SNP rs676210 was related to greater decreases in TC and LDL-C in homozygous *G* allele carriers after growth hormone replacement therapy (GHRT) [18]. Our findings have revealed that this variant (rs676210) tends to have some pleiotropic effects in patients taking 20 mg/day of atorvastatin, as shown by an elevated level of HDL cholesterol in the GG genotype compared to the AA genotype (50.71±3.40 vs. 46.63±2.76: *P* = 0.01). The study by [24] found that transcription factors such as peroxisome proliferator-activated receptor-α cause cholesterol efflux by stimulating the gene expression encoding ATP-binding cassette transporters, thereby reducing the availability of free cholesterol for storage in the form of cholesterol esters through the activity of Acyl-CoA cholesterol acyltransferase (ACAT) enzymes.

Moreover, it is necessary to perform more accurate studies focusing on the genetic variants in the pharmacogenetic analysis in order to arrive at a final conclusion on the role of genetic polymorphism in the effectiveness of drug response.

## Conclusion

In summary, the results clarified the significant role of rs676210 of the *ApoB* gene in CAD etiology, which was the *A* allele exclusively related with a protective effect against disease development while the *G* allele can be regarded as a predisposing allele. The findings support a function of (rs676210) polymorphism of the *ApoB* gene in predicting LDL-C response to 40 mg/day atorvastatin drug, thereby ApoB (rs676210, Pro2739Leu) polymorphism, specifically the AA genotype, will help to classify individuals who will benefit from atorvastatin’s decreasing effects in 40 mg/day dose.

## Data Availability

All data generated or analyzed during this study are included in this published article.

## Abbreviations

6-FAM: 6-carboxyfluorescein, acronym
AHA: American Heart Association
MI: Mayocardail infarction
ABCA1: ATP-binding cassette transporter ABCA1
TC: Total cholesterol
TG: Triglyceride
ApoB: Apolipoprotein B
ApoE: Apolipoprotein E
BMI: Body mass index
SBP: Systolic blood pressure
DBP: Distolic blood pressure
CHD: Congenital heart disease
HT: Hypertension
CVD: Cardiovascular disease
ECG: Electrocardiographic
ELISA: Enzyme-linked immune sorbent assay
GWAS: Genome-wide association study
GHRT: Growth hormone replacement therapy
HWE: Hardy-Weinberg equilibrium
HDL: High-density lipoproteins
LDL-C: Low-density lipoprotein cholesterol
NCBI: National Center for Biotechnology Information
ox-LDL: Oxidize low-density lipoprotein
RT-PCR: Real-time PCR
SNP: Single nucleotide polymorphisms
TMT: Treadmill test
VLDL: Very low-density lipoprotein
WHO: World Health Organization

## References

[1] Linton MF, Yancey PG, Davies SS, Jerome WG, Linton EF, Song WL, Doran AC, Vickers KC (2019). The role of lipids and lipoproteins in atherosclerosis. Endotext.

[2] Pahwa R, Jialal I (2018). Atherosclerosis.

[3] Yang H, Mohamed AS, Zhou SH (2012). Oxidized low density lipoprotein, stem cells, and atherosclerosis. Lipids Health Dis.

[4] World Health Organization (2016). Hearts: technical package for cardiovascular disease management in primary health care.

[5] Thaker AM, Frishman WH (2014). Sortilin: the mechanistic link between genes, cholesterol, and coronary artery disease. Cardiol Rev.

[6] Lam YW, Scott SR (2018). Pharmacogenomics: challenges and opportunities in therapeutic implementation.

[7] Zaman MM, Ikemoto S, Yoshiike N, Date C, Yokoyama T, Tanaka H (1997). Association of apolipoprotein genetic polymorphisms with plasma cholesterol in a Japanese rural population: the Shibata study. Arterioscler Thromb Vasc Biol.

[8] Gu QL, Han Y, Lan YM, Li Y, Kou W, Zhou YS, Hai XJ, Yan B, Ci CH (2017) Association between polymorphisms in the APOB gene and hyperlipidemia in the Chinese Yugur population. Braz J Med Biol Res 50(11) 10.1590/1414-431X20176613 PMC5597288 28902930

[9] Aulchenko YS, Ripatti S, Lindqvist I, Boomsma D, Heid IM, Pramstaller PP, Penninx BW, Janssens AC, Wilson JF, Spector T, Martin NG (2009). Loci influencing lipid levels and coronary heart disease risk in 16 European population cohorts. Nat Genet.

[10] Liu C, Yang J, Han W, Zhang Q, Shang X, Li X, Lu F, Liu X (2015). Polymorphisms in ApoB gene are associated with risk of myocardial infarction and serum ApoB levels in a Chinese population. Int J Clin Exp Med.

[11] Whitfield AJ, Barrett PH, Van Bockxmeer FM, Burnett JR (2004). Lipid disorders and mutations in the APOB gene. Clin Chem.

[12] Barbarash O, Gruzdeva O, Uchasova E, Belik E, Dyleva Y, Karetnikova V (2015). Dose-dependent effects of atorvastatin on myocardial infarction. Drug Des Devel Ther.

[13] Abdulfattah SY, Abdullah SJ, Alsaffar HB (2020). A study to explore the LDLR gene polymorphisms contribute to atorvastatin response in a sample of Iraqi population with atherosclerotic coronary artery disease. Karbala Int J Mod Sci.

[14] Mäkelä KM, Traylor M, Oksala N, Kleber ME, Seppälä I, Lyytikäinen LP, Sudlow C (2014). Association of the novel single-nucleotide polymorphism which increases oxidized low-density lipoprotein levels with cerebrovascular disease events. Atherosclerosis.

[15] Holvoet P, Mertens A, Verhamme P, Bogaerts K, Beyens G, Verhaeghe R, Collen D, Muls E, Van de Werf F (2001). Circulating oxidized LDL is a useful marker for identifying patients with coronary artery disease. Arterioscler Thromb Vasc Biol.

[16] Abdulfattah SY, Al-Awadi SJ, Al-Saffar HB (2020). Plasma oxidized low-density lipoprotein level and miRNA-146a gene expression, as a strong predictor for atherosclerotic coronary artery disease and its associated response to atorvastatin in a sample of the Iraqi population. Gene Rep.

[17] Wojczynski MK, Gao G, Borecki I, Hopkins PN, Parnell L, Lai CQ, Ordovas JM, Chung BH, Arnett DK (2010). Apolipoprotein B genetic variants modify the response to fenofibrate: a GOLDN study. J Lipid Res.

[18] Barbosa EJ, Glad CA, Nilsson AG, Filipsson Nystrm H, Gtherstrm G, Svensson PA, Vinotti I, Bengtsson BK, Nilsson S, Boguszewski CL, Johannsson G (2012). Genotypes associated with lipid metabolism contribute to differences in serum lipid profile of GH-deficient adults before and after GH replacement therapy. Eur J Endocrinol.

[19] Weber C, Noels H (2011). Atherosclerosis: current pathogenesis and therapeutic options. Nat Med.

[20] Teslovich TM, Musunuru K, Smith AV, Edmondson AC, Stylianou IM, Koseki M, Pirruccello JP, Ripatti S, Chasman DI, Willer CJ, Johansen CT (2010). Biological, clinical and population relevance of 95 loci for blood lipids. Nature.

[21] Ye P, Shang Y, Ding X (2003). The influence of apolipoprotein B and E gene polymorphisms on the response to simvastatin therapy in patients with hyperlipidemia. Chin Med Sci J.

[22] Chasman DI, Pare G, Mora S, Hopewell JC, Peloso G, Clarke R, Cupples LA, Hamsten A, Kathiresan S, Mälarstig A, Ordovas JM (2009). Forty-three loci associated with plasma lipoprotein size, concentration, and cholesterol content in genome-wide analysis. PLoS Genet.

[23] Voora D, Shah SH, Reed CR, Zhai J, Crosslin DR, Messer C, Salisbury BA, Ginsburg GS (2008). Pharmacogenetic predictors of statin-mediated low-density lipoprotein cholesterol reduction and dose response. Circ Cardiovasc Genet.

[24] Parihar SP, Guler R, Brombacher F (2019). Statins: a viable candidate for host-directed therapy against infectious diseases. Nat Rev Immunol.

